# IS*Ec69*-Mediated Mobilization of the Colistin Resistance Gene *mcr-2* in *Escherichia coli*

**DOI:** 10.3389/fmicb.2020.564973

**Published:** 2021-01-12

**Authors:** Yu-Zhang He, Teng-Fei Long, Bing He, Xing-Ping Li, Gong Li, Liang Chen, Xiao-Ping Liao, Ya-Hong Liu, Jian Sun

**Affiliations:** ^1^National Risk Assessment Laboratory for Antimicrobial Resistance of Animal Original Bacteria, South China Agricultural University, Guangzhou, China; ^2^Guangdong Provincial Key Laboratory of Veterinary Pharmaceutics Development and Safety Evaluation, South China Agricultural University, Guangzhou, China; ^3^Hackensack Meridian Health Center for Discovery and Innovation, Nutley, NJ, United States

**Keywords:** transposition mechanism, transposition sites, *mcr-2* gene, IS*Ec69*, colistin resistance

## Abstract

**Objectives:**

The emergence of mobile colistin resistance genes has compromised the efficacy of the last resort antibiotic, colistin, in clinical treatment. The *mcr-2* gene was first identified in Belgium in association with the insertion sequence IS*Ec69*. However, the molecular mechanisms of *mcr-2* mobilization are not well understood.

**Methods:**

To further explore the mobilization of *mcr-2* gene via IS*Ec69*, we constructed a conjugative plasmid that carries an intact composite transposon Tn7052. Transposition assays were performed by conjugation, the transposition sites were characterized by arbitrary primed PCR and DNA sequencing.

**Results:**

In this study, we experimentally demonstrated that *mcr-2* could be mobilized as a composite transposon Tn7052 and its transposition generated 8-bp AT-rich duplications in the host genome.

**Conclusion:**

These results indicate that *mcr-2* gene could be mobilized by IS*Ec69*, the current investigations provide mechanistic insights in the transposition of *mcr-2*.

## Introduction

The global dissemination of multidrug resistant bacteria, especially extended-spectrum cephalosporin and carbapenem resistant “ESKAPE” pathogens (*Enterococcus faecium, Staphylococcus aureus, Klebsiella pneumoniae, Acinetobacter baumannii, Pseudomonas aeruginosa*, and *Enterobacter* spp.) become a significant threat to public health (Deslouches et al., 2015). Meanwhile, the identification of mobile colistin resistance genes (*mcr*) has compromised the efficacy of colistin as the last-resort antibiotics against these pathogens.

The *mcr-2* is a novel plasmid-mediated colistin resistance gene that is 81% identical to *mcr-1* and is associated with IS*Ec69* of the IS*1595* superfamily. The *mcr*-2 was initially reported on a 35 kb IncX4 plasmid, pKP87-BE, flanked by directly oriented copies of IS*Ec69* (Partridge, 2017). Currently, the actual and reliable experimental data in regard to mechanism underlying the *mcr-2* gene horizontal transfer are rarely reported. The transfer of antimicrobial resistance genes has been found to be catalyzed by specific mobile elements such as insertion element and integron. For instance, IS*Apl1*, originally found in *Actinobacillus pleuropneumoniae*, is located upstream of the *mcr-1* gene in the first described *mcr-1*-harboring IncI2 type plasmid pHNSHP45 (Liu et al., 2015; Ye et al., 2016) and is able to mediate *mcr-1* mobilization (Poirel et al., 2017; He et al., 2019). Similarly, IS*Ec69*, encoding a 217-amino-acid-long DDE-type transposase, belongs to the IS*1595* family and is flanked by two inverted repeats, which share 16/19 nucleotide identity to inverted repeats from the IS*1016C2* transposase. Directly downstream of *mcr-2* is the *pap2* (*ORF*) gene, encoding a PAP2 membrane-associated lipid phosphatase with 41% identity with *Moraxella osloensis* phosphatidic acid phosphatase. Tn*7052*(IS*Ec69-mcr-2-ORF-*IS*Ec69*) forms a composite transposon structure, and we suspected *mcr-2* may be mobilized through transposition.

The aim of the current study was to experimentally determine whether IS*Ec69* could mobilize the *mcr-2* gene and to identify the integration sites on bacterial genome. Preliminary experiments showed that the appearance of a circularized intermediate might accelerate the dissemination of *mcr-2* (Sun et al., 2017). However, the mechanism of *mcr-2* transfer has not yet to be studied, especially for its transposition. In this work, we focused on providing experimental evidence for the specific transposition of *mcr-2* as well as the host transfer via conjugation and subsequent transposition into the host chromosome.

## Materials and Methods

### Strains

*E. coli* MG1655 (wild-type) and *E. coli* MG1655 (*recA*::*Km*) strains were used as recipients for transposition experiments as previously described (Gerdes et al., 2003). *E. coli* strain WM3064 (Dehio and Meyer, 1997) is a diaminopimelic acid (DAP) auxotroph strain, containing RP4 plasmid transfer machinery (McLaughlin and Ahmad, 1986) and the *pir* (Chen et al., 1998) plasmid maintenance sequence with an R6K (Rakowski and Filutowicz, 2013) *ori*. This strain was also used as a host for suicide plasmid bearing Tn*7052*. The strains and plasmids used in this study are listed in Table 1.

**TABLE 1:** Strains and plasmids used in this study.

	**Description**	**References**
*E. coli* MG1655 (wild-type)	K-12 strain F–λ–ilvG rfb-50 rph-1	Gerdes et al., 2003
*E. coli* MG1655(*recA*::*Km*)	K-12 strain F–λ–ilvG rfb-50 rph-1 recA^–^	Gerdes et al., 2003
*E. coli* WM3064	RP4(tra) in chromosome, DAP^–^	Dehio and Meyer (1997)
pSV03	CmR, replication origin from *E. coli* plasmid R6K; requires the R6K initiator protein pir for replication	Lab storage
pCVD442	R6K ori, mobRP4, *bla*, *sacB*	Donnenberg and Kaper (1991)
pJS05	Suicide plasmid (R6K replication origin) contains Tn*7052*	This study
pJS05-TraJ	Suicide plasmid [R6K replication origin, RP4(tra)] contains Tn*7052*	This study
pUC57-*mcr-2*	Engineered plasmid containing *mcr-2* gene which is flanked by IS*Ec69* on both sides	This study

### Plasmid Construction

We firstly constructed the suicide plasmid pJS05-Traj, containing the Tn*7052* cassette, RP4_*oriT*_ fragment from pCVD442 (Philippe et al., 2004) and R6K replication origin which relies on π protein, encoded by the *pir* gene. This suicide plasmid will only survive in a bacterial host with the *pir* gene (e.g., *E. coli* WM 3064), but won’t be able to replicate in other hosts. In brief, we synthesized a cassette in which the *mcr-2 and orf* genes are flanked by IS*Ec69* on both sides and cloned into the vector pUC57, generating the plasmid pUC57-*mcr-2*. R6K-*Bam*HI and R6K-*Eco*RI primers (Table 2) were used to PCR amplify the backbone using pSV03 (He et al., 2019) as a template which contained the conditional replication origin R6K and Cm^*R*^ (chloramphenicol) resistance. The amplicon was sub-cloned into the *Bam*HI and *Eco*RI restriction sites in pUC57 that contained Tn*7052* to obtain recombinant plasmid pJS05 (Supplementary Figure 1A). The plasmid was electroporated into strain *E. coli* WM 3064 and clones were selected on Luria Bertani (LB) agar plates supplemented with 25 μg/ml chloramphenicol. The integrity of both IS*Ec69* and *mcr-2* was confirmed by DNA sequencing. Traj-F and Traj-R primers (Table 2) were used to amplify RP4_*oriT*_ fragment using pCVD442 as a template by PCR. The RP4_*oriT*_ fragment was ligated into the *Sal*I and *Eco*T22I sites of pJS05 giving rise to recombinant plasmid pJS05-Traj (Supplementary Figure 1B). The plasmid was electroporated into *E. coli* WM 3064 and selected on LB agar plates supplemented with 25 μg/ml chloramphenicol. The integrity of pJS05-Traj was confirmed by whole plasmid sequencing.

**TABLE 2:** Primers used in this study ±.

**Primers**	**Sequence (5′→ 3′)**
R6K-*Bam*HI	TACGCGGATCCGTTGATCGGCACGTAAGAGG
R6K-*Eco*RI	TACGCGAATTCCCATGTCAGCCGTTAAGTGT
Traj-F	TACGCGTCGACTCGTCTGGAAGGCAGTACACCTT
Traj-R	TACGCATGCATCCAGTCGGTAGATATTCCAC
AB1	GGCCACGCGTCGACTAGTACNNNNNNNNNNACGCC
AB2	GGCCACGCGTCGACTAGTACNNNNNNNNNNCCTGG
AB3	GGCCACGCGTCGACTAGTACNNNNNNNNNNCCTCG
ABS	GGCCACGCGTCGACTAGTA
ISEc69-F1	CCAAAGCCAAGACATTGATGCC
ISEc69-F2	TTGGTCGCAATCCAAACGCA
IS69-R	CTGATCTTGCGGTGACTTCA

*± All the primers were designed in this study and restriction sites are underlined.*

### Transposition Assays

Transposition of the recombinant plasmids was examined using *E. coli* WM3064/pJS05-Traj as a donor and *E. coli* MG1655 (wild-type) and *E. coli* MG1655 (*recA*::*Km*) as recipients. Cells were cultured to mid-log phase (OD_600_ 0.5) at 37°C in LB plus 300 μM DAP for donor, and 100 μL of each a donor and recipient were mixed and vortexed. The cells were then collected by centrifugation (16,100 × *g* for 30 s at 25°C) and suspended in 100 mL fresh LB containing 300 μM DAP and spread on LB agar plates containing 300 μM DAP and incubated at 37°C for 12 h. The cells were then recovered from the plates and suspended in 1 mL LB and aliquots were plated on LB agar containing 2 μg/ml colistin (without DAP) to select for transposons. Since the *E. coli* WM3064 is an auxotrophic strain whose growth relies on the supplementation of diaminopimelic acid (DAP) in the medium (Wang et al., 2015), and the plasmid pJS05-Traj can’t replicate in *E. coli* MG1655, the colonies that grow on LB agar plates containing 2 μg/ml colistin (without DAP) were likely the transposons. The presence of the full-length Tn*7052* that integrated into the *E. coli* MG1655 genome was confirmed using PCR with primers ISEc69-F1 and IS69-R (Table 2). Transposition frequencies were calculated as the ratio of transposition events and the number of transformed cells in triplicate. The Pictogram program^1^ was used to illustrate the relative positions of transposition sites.

### Arbitrary Primed PCR(AP-PCR) for Rapid Characterization of Transposon Insertion Sites

Transposon insertion sites in strain *E. coli* MG1655 (*recA*::*Km*) were identified by screening of 30 randomly selected colonies that were prepared from overnight cultures. DNA was extracted using the TIANamp Bacteria DNA Kit (Tiangen, Dalian, China). The right-side of the Tn*7052* in the genome was confirmed by AP-PCR (Das et al., 2005). Briefly, DNAs from transposons were used as templates for the first-round PCR reactions. The arbitrary primers differed from their 3′ pentameric sequences (ABS1 to ABS3; Table 2) were paired in separate PCR reactions with the transposon-specific primer ISEc69-F1. The products of these reactions served as templates for second-round PCR reactions employing a nested transposon primer ISEc69-F2 and a primer composed of the common 5′ region from ABS1 to ABS3 (ABS; Table 2; Das et al., 2005). The identities of PCR products and associated genomic flanking regions were determined by DNA sequencing (Supplementary Figure 2). The left-side DNA sequences of Tn*7052* in the genome were confirmed by standard PCR using primers TF_1_∼TF_23_ paired with CR-2U (Supplementary Table 1), respectively.

## Results

### The Transposition Frequencies of pJS05-Traj Into Two *E. coli* Strains

We identified the transposition abilities of the Tn*7052* cassette by cloning it into a suicide plasmid that was then electroporated into the *pir*^+^ strain *E. coli* WM3064. This plasmid was transferred to recipient strains *E. coli* MG1655 (wild type) and *E. coli* MG1655 (*recA*::*Km*) by conjugation. The survival of the conjugants was contingent upon transposition of the selectable marker (*mcr-2*) into the host genome. The transposition frequencies of pJS05-Traj into *E. coli* MG1655 (wild type) and *E coli* MG1655 (*recA*::*Km*) strains were 4.09 × 10^–6^ and 3.53 × 10^–6^ per transformed cell, respectively (Table 3).

**TABLE 3:** Transposition frequencies of *mcr-2* in two *E. coli* strains.

**Suicide plasmid**	**Target**	**Frequency**		
		**Incubation drug**	**Mean**	**Range**
pJS05-Traj	*E. coli* MG1655(wild-type)	2 μg/ml colistin	4.09 × 10^–6^	4.21 × 10^–6^ - 3.97 × 10^–6^
	*E. coli* MG1655(*recA*::*Km*)	2 μg/ml colistin	3.53 × 10^–6^	3.84 × 10^–6^ - 3.22 × 10^–6^

### The Transposition Sites Preference of Tn*7052* in the *E. coli* MG1655 (*recA*::*Km*) Genome

**FIGURE 1:**
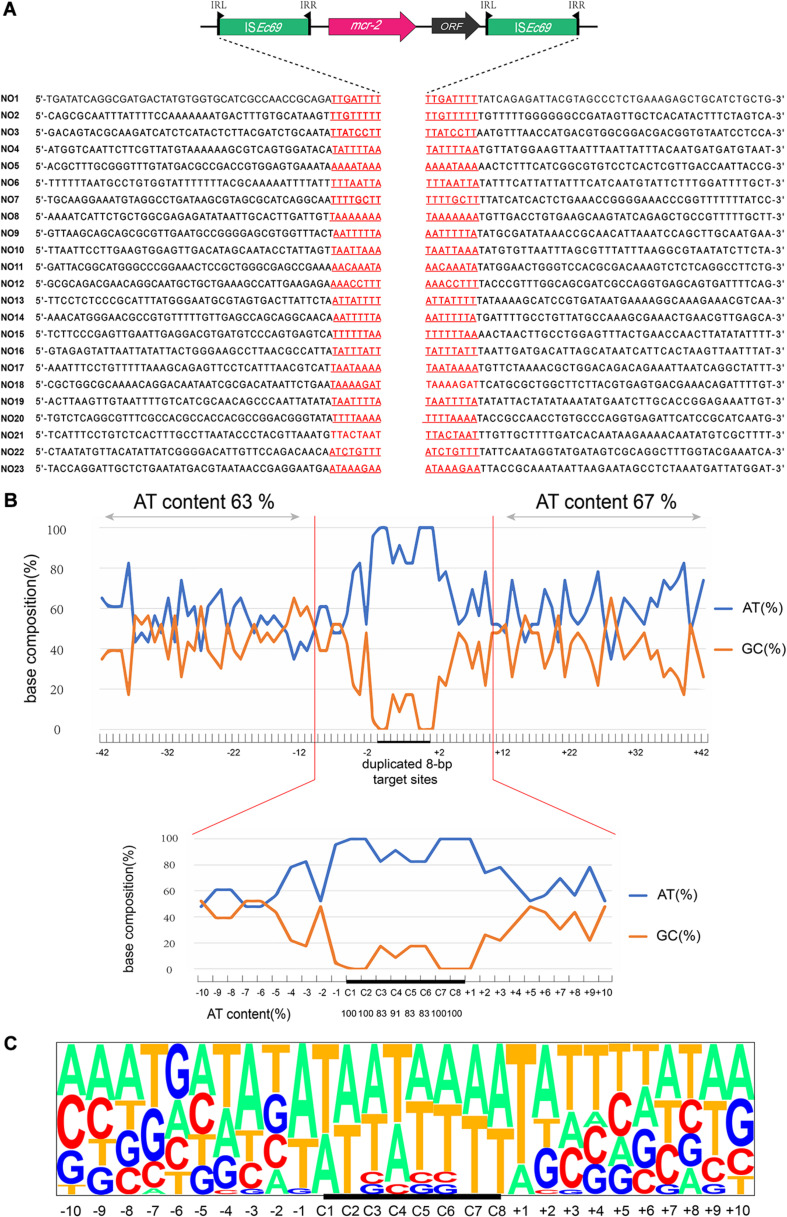
Target site analyses of Tn*7052* transposons. **(A)** Molecular characterization of 23 transposition events of Tn*7052* transposons in *E. coli* MG1655 (*recA*::*Km*). The duplicated 8-bp target sites are underlined in the context of the surrounding 42 nucleotides upstream and downstream from the target sites. **(B)** Statistical analyses of the 23 transposition sites. The percentage of AT and GC at each position from 42 nucleotides upstream to 42 nucleotides downstream of the target site are shown. The 8-bp duplicated target site (C1 to C8) are indicated by black bars. The AT and GC percentages of regions spanning positions –42 to –2 and positions +2 to +42 and that of the region spanning positions –2 to 2 are indicated in the upper and lower graphs, respectively. Relative nucleotide frequencies at each target site deduced from the 23 experimental transposition events shown in **(A,C)**.

AP-PCR-based analysis identified 23 Tn*7052* integration sites in the recipient chromosome among the 30 colonies. These events generated 8-bp sequence duplications at AT-rich regions with a high preference for insertion between T and A. The average AT contents at the 40 bp adjacent regions upstream and downstream of the 8-bp target sites were 63 and 67%, respectively (Figure 1A). In addition, the AT content at the target sites were 100% at positions in C1, C2, C7, and C8 and ranging from 83 to 91% at positions C3, C4, C5, and C6. Transposition outside the sequence duplicated target site (+2 to +10) ranged from 35 to 83% (Figure 1B).

### The Transposition Sites Position in the *E. coli* MG1655 (*recA*::*Km*) Genome

We further examined these 23 transposition events and calculated the relative frequencies of AT/GC and plotted for the region extending from 50 nucleotides upstream to 50 nucleotides downstream from the duplicated 8-bp target site (Figure 1C). In addition, the genomic locations of Tn7052 integration sites were further analyzed. The Tn7052 sites were all located in different single genes in the recipient chromosome (Supplementary Table 2).

## Discussion

In this report, we illustrated a working model for the transposition of a *mcr-2*-containing cassette from a plasmid containing a unique transposon-like region. The *mcr-2* gene conferring colistin resistance was identified on a plasmid vector (Xavier et al., 2016). However, its mobilization mechanism has not been confirmed.

Although *mcr-2* gene has not yet spread in the world, it only has only been identified once, on a IncX4 conjugative plasmid (Partridge, 2017). It is very important to study its transmission and transposable mechanism in advance. The transposition of the MCR-2 protein encoding gene is closely linked to IS*Ec69*. Here, we used R6K *ori* to enable replication of a pJS05-Traj bearing Tn*7052* element in *E. coli* WM3064. In addition, the origin of conjugative transfer (RP4_*oriT*_) was inserted to allow efficient transfer of the suicide plasmid to other *E. coli* hosts. This process was successful and implicates conjugational transfer as an effective mobilization method for *mcr-2* into recipient strain chromosomal locations mediated by IS*Ec69* transposition. Interestingly, Tn*7052* insertion sites duplicated an 8 bp AT-rich target site. In prokaryotic, the AT-rich region is generally the replication origin of bacterial chromosomes and plasmids (Rajewska et al., 2012). The transposition in the replication origin of bacterial chromosomes and plasmids may have a negative impact on bacterial and plasmid replication, especially on plasmid copy number and its stability. This may be one of the reasons for the low prevalence of *mcr-2*.

The 23 precise transposition sites were characterized by AP-PCR that is a lightweight and reliable tool for IS detection and transposon insertion site identification in bacterial genomes. Compared with previous digestion and inverse PCR strategies (Green and Sambrook, 2019), the method might be more convenient and efficient, and the cost is comparatively lower as well. The method is simple to perform and can be used in any laboratory that is equipped with PCR equipment for identifying transposase insertion sites in bacterial genomes. To further characterize the distribution of IS*Ec69* mediated *mcr-2* transposition, we determined the insertion sites for 23 transposon events in *E. coli* MG1655(*recA*::*Km*) genome. We found that 11 Tn*7052* transposition sites were randomly located into non-essential genes and 12 were inserted between two non-essential genes in the bacterial chromosome (Supplementary Table 2). This study only finds insertions in non-essential genes, maybe since insertions into essential genes are deleterious and may negatively impact growth. Therefore, they cannot be selected on plates and are unable to be identified.

Taken together, these results indicated that *mcr-2* most likely was mobilized by an IS*Ec69* composite transposon, generating an 8-bp DR at TA-rich sites. Our work demonstrated that IS*Ec69* possessed the ability to transpose *mcr-2* in *E. coli* that may act as a reservoir for the *mcr-2* gene and contribute to its dissemination among different bacterial species. This is especially important for clinically relevant bacterial species such as *Enterobacteriaceae* family members. Compared with *mcr-1*, the prevalence of *mcr-2* is relatively low. It may relate to the transposable capacity of IS*Ec69*. The transposition experiments in this study were designed to investigate the mechanisms by which insertion sequences IS*Ec69* mediate the transfer of *mcr-2* gene, including the insertion site’s preference and the transposition efficiency. Understanding these knowledges could help control the spread of *mcr-2* gene.

Duo to very low prevalence of *mcr-2* plasmid-mediated colistin, it is speculated that the distribution of *mcr-2* between European countries might be related to geography or differences in veterinary practices between these regions, but further research is needed. In the future, more intensive and concrete work will focus on the other routes of *mcr-2* gene transmission so as to elucidate pathways for blocking the spread of the *mcr-2*.

Accession number: The nucleotide sequence of Tn*7052* has been deposited in GenBank under accession numbers: MW251710.

## Data Availability Statement

The raw data supporting the conclusions of this article will be made available by the authors, without undue reservation. The authors declare that, all the experiments were performed strictly according to the guidelines of WHO (WHO-DURC). All samples including recombinant strains, plasmid, and their relevant storages are properly maintained during and after the research. These materials are currently kept with high biosafety standard and cannot be accessed without certain permission. The authors guaranteed all materials employed in this study will be only used for scientific purposes and no risk on spreading the antibiotic resistance to the naturally existing microorganisms.

## Author Contributions

JS designed this project. Y-ZH, T-FL, and BH performed the experiments. Y-ZH, T-FL, X-PLi, and JS analyzed the data. Y-ZH and GL made the figures. Y-ZH wrote this manuscript. JS, LC, and X-PLa edited and revised the manuscript. Y-HL coordinated the whole project. All authors contributed to the article and approved the submitted version.

## Conflict of Interest

The authors declare that the research was conducted in the absence of any commercial or financial relationships that could be construed as a potential conflict of interest.

## Footnotes

1 http://genes.mit.edu/pictogram.html
