# Supplementary material for: ISEc69-Mediated Mobilization of the Colistin Resistance Gene mcr-2 in Escherichia coli
Source: Front Microbiol. 2021 Jan 12;11:564973. doi: 10.3389/fmicb.2020.564973 (PMC7835479; doi:10.3389/fmicb.2020.564973)
Supplement: Supplementary file 1 [file Data_Sheet_1.docx]

**Supplementary materials**

Supplementary Figures

**Fig S1**


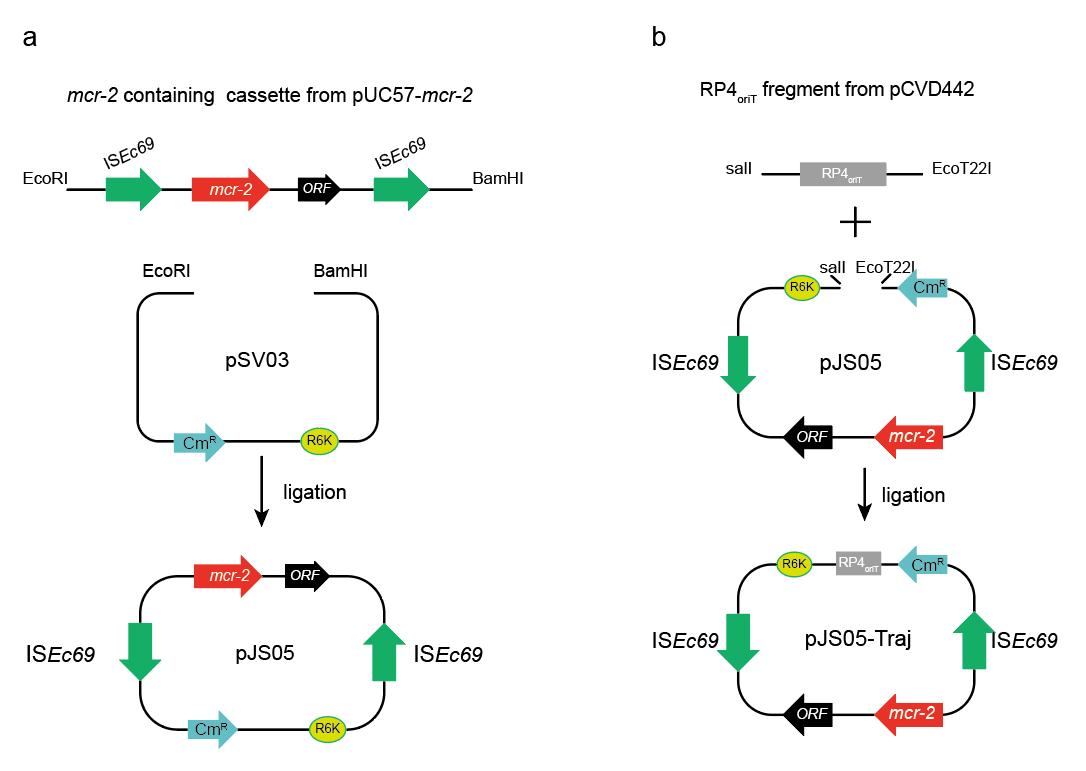


**Fig S2**


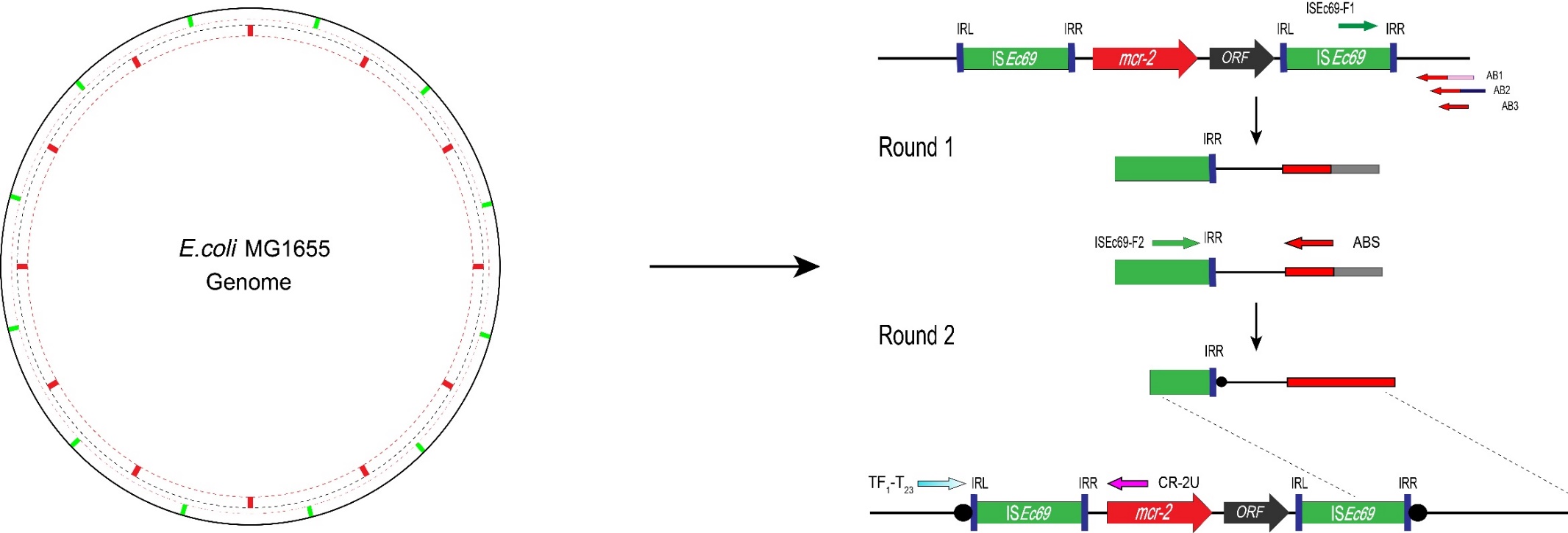


**Fig S2｜**Schematic for determination of transposition sites by arbitrary primed PCR (AP-PCR). Primer ISEc69-F1 was paired in a separate first-round PCR reaction with an arbitrary primer (AB1 or AB2 or AB3), depicted as solid and striped red arrows, respectively. Low annealing temperatures were employed to allow arbitrary primers to bind to many sites. Products from the first-round reactions were then used as templates for second-round reactions employing nested, sequence-specific primers (green arrows) paired with the ABS primer (red arrows) that were identical to the 5＇sequence of the first-round arbitrary primers. The left side DNA sequence of this structure was confirmed using second-round amplicons that were generated using primers TF_1_ to TF_23_ paired with primer CR-2U, separately. The inverted repeats (IRL and IRR) are represented as blue vertical bars and direct repeats (DR) as black oval.

Supplementary tables

**Table S1**

| primers | Sequence ( ­­5＇→ 3＇) | References |
| --- | --- | --- |
| CR-2U | GACCAACACACTTGGCAACA | This study |
| TF1 | GATTATGCACCTGCAATTCGC | This study |
| TF2 | CGTCAGATGAGTTTGTGCAT | This study |
| TF3 | GCTGCTGTTGGTTTGTTTAC | This study |
| TF4 | TGGTTGCCTGACTCACAAAA | This study |
| TF5 | TGATTGCTTTTACGGGCGCA | This study |
| TF6 | CTTCTTGAAACCACCGCAAA | This study |
| TF7 | AGGAACGCTATCACCGTTTCC | This study |
| TF8 | AACCGTCACCATGCCATTAC | This study |
| TF9 | TCTGAATCCGAACTTACGGT | This study |
| TF10 | CAATGGCCCCTGGATGAAAG | This study |
| TF11 | TGACGAACATTTCGTGCTGG | This study |
| TF12 | CCGATTCTGCTTAACGGTCC | This study |
| TF13 | TCTCCCGGCAACATTTATTGCC | This study |
| TF14 | ATAGTCAGGGTCAGGTGCAG | This study |
| TF15 | TGAGGTCAGTTTAAAGGCGGT | This study |
| TF16 | ACAGCGATAAGTGTACCGAATG | This study |
| TF17 | GACTTGTGCAACAGTTCGCG | This study |
| TF18 | AGCAGCAATAAACCGCCTAC | This study |
| TF19 | GTTGCTCGTTTCTGTCCAGC | This study |
| TF20 | ACGCTTCGTGCAAATGATGA | This study |
| TF21 | GGCCTTTAGTCGTGGGGATT | This study |
| TF22 | TTGGTGCGAAATTACGGCAG | This study |
| TF23 | TGCGAAGGTCGCAATATGCT | This study |

**Table S2**:Insertion sites of Tn*7052* in the *E.coli* MG1655 (*recA*∷*Km*) genome±­­­­­

| Transposition  events | Insertion  sites | Function |
| --- | --- | --- |
| NO1 | *ynbC* | Putative lipase/methylase |
| NO2 | *ycdT* | Diguanylate cyclase |
| NO3 | *yncL/patD* | stress-induced small inner membrane enterobacterial protein/gamma-aminobutyraldehyde dehydrogenase |
| NO4 | *yfgG/ yfgF* | uncharacterized protein/ cyclic-di-GMPphosphodiesterase, anaerobic |
| NO5 | *yjiL* | putative ATPase, activator of (R)-hydroxyglutaryl-CoA dehydratase |
| NO6 | *ygeN/ ygeK* | unknown/unknown |
| NO7 | *trpS/ yhfZ* | tryptophanyl-tRNA synthetase |
| NO8 | *yedA/ yedI* | amino acid exporter for phenylalanine, threonine/ DUF808 family inner membrane protein |
| NO9 | *yafP* | GNAT family putative N-acetyltransferase |
| NO10 | *frc* | formyl-CoA transferase, NAD(P)-binding |
| NO11 | *frdA* | anaerobic fumarate reductase catalytic and NAD/flavoprotein subunit |
| NO12 | *rtcR* | sigma 54-dependent transcriptional regulator of rtcBA expression |
| NO13 | *yigE* | DUF2233 family protein |
| NO14 | *ecpR* | putative transcriptional regulator for the ecp operon |
| NO15 | *ecpA/ ecpR* | ECP pilin/ putative transcriptional regulator for the ecp operon |
| NO16 | *yfhL/ shoB* | putative 4Fe-4S cluster-containing protein/ toxic membrane protein |
| NO17 | *ypdA/ alaC* | sensor kinase regulating yhjX; pyruvate-responsive YpdAB two-componentsystem/glutamate-pyruvate aminotransferase; glutamic-pyruvic transaminase (GPT); alanine transaminase |
| NO18 | *yqgA* | DUF554 family putative inner membrane protein |
| NO19 | yiaU/yiaT | putative DNA-binding transcriptional regulator/ putative outer membrane protein |
| NO20 | *dgt* | deoxyguanosine triphosphate triphosphohydrolase |
| NO21 | *ygaQ/csiD* | unkown/carbon starvation protein |
| NO22 | *trxC/yfiF* | thioredoxin 2/ putative methyltransferase |
| NO23 | *acrB/tomB* | multidrug efflux system protein/ Hha toxicity attenuator; conjugation-related protein |

± All insertion sites were located in nonessential genes.
